# Differences in gray matter volume in episodic migraine patients with and without prior diagnosis or clinical care: a cross-sectional study

**DOI:** 10.1186/s10194-021-01340-5

**Published:** 2021-10-23

**Authors:** Shana A.B. Burrowes, Olga Goloubeva, Michael L Keaser, Jennifer A. Haythornthwaite, David A. Seminowicz

**Affiliations:** 1 Section of Infectious Diseases, Department of Medicine, Boston University School of Medicine, 02218 Boston, MA USA; 2 Department of Neural and Pain Sciences, School of Dentistry, University of Maryland Baltimore, 21201 Baltimore, MD USA; 3 Center to Advance Chronic Pain Research, University of Maryland Baltimore, 21201 Baltimore, MD USA; 4 Department of Epidemiology and Public Health, School of Medicine, University of Maryland Baltimore, 21201 Baltimore, MD USA; 5 Greenebaum Comprehensive Cancer Center, University of Maryland, University of Maryland Baltimore, 21201 Baltimore, MD USA; 6 Department of Psychiatry and Behavioral Sciences, Johns Hopkins University School of Medicine, Baltimore, MD USA; 7 Boston University School of Medicine, 801 Massachusetts Avenue Room 2004, MA 02118 Boston, USA

**Keywords:** Gray matter volume, Treatment naïve, Prior care, Depression, Stress, Migraine, Headache, MRI

## Abstract

**Background:**

Migraine sufferers face difficulties getting appropriate care and treatment. Migraine is associated with reduced gray matter volume (GMV) in several brain regions, which could be related to various clinical characteristics of the disorder.

**Objectives:**

To examine differences in GMV in migraine patients with and without prior clinical care for migraine and examine differences in migraine clinical variables, psychosocial symptoms and their relationship with GMV.

**Methods:**

We utilized the baseline MRI scan and psychosocial symptom questionnaires from a longitudinal randomized controlled trial. Prior care of migraine was determined by diagnosis by a medical practitioner or prescription of migraine specific medication.

**Results:**

117 patients were included in the study. Patients without prior care (*n*=23) had reduced GMV in the right dorsal medial prefrontal cortex (dMPFC) relative to patients who had prior care (*p*=0.034, FWE corrected). Both patient groups had reduced GMV compared to healthy controls (*n*=36). Patient groups did not differ in headache clinical variables. Regardless of care status, increasing scores on the stress (Perceived Stress Score) and depression questionnaires (Patient Health Questionnaire) were associated with increased GMV in the dMPFC.

**Conclusions:**

Clinical care may impact GMV in migraine patients. Patients may need different treatment options to address this baseline deficit.

**Trial registration:**

NCT02133209.

**Supplementary Information:**

The online version contains supplementary material available at 10.1186/s10194-021-01340-5.

## Introduction

Migraine often goes undiagnosed and migraineurs face difficulties in ascertaining appropriate care and treatment options. [1, 2] Nationwide studies in the US have shown that among persons with episodic migraine (EM), only 45.5 % had received a medical consultation in the preceding year, and of those 86.7 % received a diagnosis of migraine. Furthermore, of those migraineurs who are in need of clinical care only one quarter successfully achieved the minimum appropriate care. [3] These treatment patterns are not unique to the US. The Eurolight project comprising 10 European countries, reported that in population-based samples the proportion of patients who had seen a doctor ranged between 9.5 and 18 % and an even smaller proportion (3.1-15 %) were prescribed migraine specific abortive medication (Triptans). [4].

In the American Migraine Prevalence and Prevention Study (AMPP) it was found that there were three main steps needed to attain minimum care: appropriate medical consultation, accurate diagnosis and effective treatment. [3] Though barriers exist at each level, the greatest occur at the level of seeking care where predictors for consultation are, having access to health insurance, and headache related variables such as headache related disability and pain intensity. [3].

The need to overcome the aforementioned barriers is extremely important for several reasons, the most important being to reduce the risk of migraine patients transitioning to more chronic and severe forms of the disease. Ineffective treatment of episodic migraine is just one of the risk factors for new onset chronic migraine (CM). [5] The prevailing theory is that as a patient experiences more frequent headache attacks there is prolonged activation of neuronal networks that are involved in pain processing during attacks and through neuroplastic mechanisms have lower thresholds for subsequent attacks. [5–7] It is also hypothesized that ineffective treatment leads to longer exposures to pain which increase the risk of CM. [5].

Gray matter volume (GMV) in motor/premotor, prefrontal, cingulate, posterior parietal, and orbitofrontal cortices have been negatively correlated with headache severity, frequency, duration, and pain intensity. [8–10] The right anterior cingulate cortex (ACC) and bilateral insula have been associated with headache frequency in episodic migraine patients. [8, 11] Chen et al. also reported decreased GMV in the left superior frontal gyrus (SFG) in migraine patients (both EM and CM) when compared to tension type headache patients, suggesting that differences in the SFG are specific to headache type. [11] Additionally greater pain intensity and disease duration have been associated with reduced GMV in the bilateral posterior and the left anterior insula. [12] Hubbard et al. further found reduced cortical thickness in the bilateral dorsolateral prefrontal cortex (DLPFC) associated with increased attack frequency and longer disease duration [12], providing further evidence that structural changes to these areas are associated with severity of disease.

However, there has not been any investigation into the pathophysiological association between ineffective or lack of treatment and migraine and it is clear that this is a gap that needs to be addressed. In this study we examined GMV differences between two groups of EM patients, those who have received care for their migraine and those who have not. We hypothesized that at baseline GMV will differ in specific pain and affective processing areas such as the DLPFC, insula and ACC based on prior diagnosis/care of migraine at baseline. Exploratory analysis examined the relationship between psychosocial symptoms and GMV.

## Methods

### Study design and population

Participants included in this cross-sectional analysis of GMV were enrolled in the MRI Outcomes of Mindfulness Meditation for Migraine clinical trial (NCT02133209). The Johns Hopkins School of Medicine and University of Maryland Baltimore Institutional Review Boards approved the study. All participants provided written consent. Participants were enrolled from June 2014 to February 2017. Participants were aged 18-65 and were not eligible if they had a history of mindfulness meditation practice. All migraine patients were episodic migraineurs (EM) as defined by the International Classification of Headache Disorders Criteria-II for migraine. [13] Migraine patients were prospectively recruited from patient registries at headache clinics at John’s Hopkins and University of Maryland Medical Center and via print, electronic and radio media. Migraine patients completed 28-day headache diaries at enrollment and were eligible if they reported 4-14 headache days at baseline. Screening and intervention visits for migraine patients were conducted at Johns Hopkins Bayview Medical Center. The study also enrolled 39 healthy pain free controls, matched to the migraine patients on age (±5 years), sex, body-mass index (BMI) (±5), education (college and no college) and race. The main outcomes of the clinical trial have been published elsewhere. [14].

Neuroimaging visits for participants occurred at the University of Maryland Baltimore. At each visit participants underwent an MRI, as well as completed several psychosocial and quality of life questionnaires. Patients provided headache diary data and psychosocial and quality of life questionnaires at three time points, but this study only utilizes baseline data. Headache diaries were completed electronically (online via a link sent through email) and collected detailed information on headaches over a 28 day period. Information on the quality of the headache including type of pain, headache duration and associated symptoms were collected. These analyses utilized diagnosis information, MRI, headache diary and questionnaire data at baseline. Participants who were enrolled but did not complete MRI visits, have unusable MRI data or missing data on prior clinical care and treatment for migraine were not included in this study.

### Assessment of prior clinical care

Migraine diagnosis or clinical care prior to enrollment in the study was established by detailed interview and health history form (HHF) during the baseline screening at Johns Hopkins. The HHF form collected information on current and past medical diagnoses and problems, prescription and over the counter medications including doses and life style factors such as smoking, drinking and exercise. Patients completed a separate screen about history of their migraine including but not limited to prior diagnoses and treatment information for migraine headaches. Based on information from their HHF and migraine screening session, patients were categorized into two groups (prior care and no prior care for migraine) before study enrollment. Categorization was achieved using patient report of diagnosis from a medical practitioner (specialist or general practitioner) or prescription of migraine specific medication for which the intended use was migraine prevention or treatment. Patients with either a diagnosis or prescribed treatment were categorized as having prior care. Patients who did not have a history of migraine diagnosis or prescribed migraine medication were classified as not having any prior care for migraine. However, all patients were officially diagnosed with migraine at enrollment by a neurologist at Johns Hopkins using ICHD-II criteria.

### MRI procedures

All baseline MRI scans were performed from September 2014 through March 2017 and completed on a Siemens Tim-Trio 3T MRI scanner using a 32-channel head coil. This study utilized scan data from the T1 MPRAGE. Acquisition parameters for the T1 MPRAGE were as follows: repetition time (TR) 2300ms, echo time (TE) 2.98ms, slice thickness 1mm, field of view (FOV) 256mm, flip angle 9°, voxel sixe (1 × 1 × 1mm).

### MRI preprocessing and analysis methods

We used voxel based morphometry (VBM) to assess differences in GMV in episodic migraine patients with and without prior clinical care/treatment for migraine at baseline. [15] All images were realigned to the anterior-posterior commissure in Statistical Parametric Mapping (SPM12) (https://fil.ion.ucl.ac.uk/spm/) prior to pre-processing. The computational anatomy (CAT12.1, r1250) toolbox within SPM12 was used to assess VBM of GMV in both patient groups. [16] Using the cross sectional segmentation pipeline in CAT12, the baseline structural T1-weighted images were spatially normalized to Montreal Neurological Institute (MNI) space (resampled to a voxel size of 1.5mm x 1.5mm x 1.5mm), segmented into gray matter (GM), white matter (WM) and cerebrospinal fluid (CSF). Scans were preprocessed with an absolute threshold mask of 0.1. This threshold excluded voxels with less than 10 % probability of being gray matter. Finally, images were smoothed with an 8mm Gaussian Kernel prior to analysis.

### Explicit mask analyses

To assess the difference in GMV between EM patients with and without prior clinical care we conducted explicit mask and whole brain analyses. The explicit mask comprised the bilateral SFG, DLPFC, insula and ACC. The inclusion of these regions in the explicit mask was based on their relationship with migraine clinical characteristics and were included in our preregistered analysis plan (https://clinicaltrials.gov/ct2/show/NCT02133209). All regions included in the analysis were made from the Atlas of Intrinsic Connectivity of Homotopic Areas. [17] Given no a-priori hypotheses about laterality all regions were made for both the left and right sides of the brain. The regions were combined using the “-add” function in FSLMATHS and displayed for inspection on a standard brain in MNI space (Additional File 1).

### SPM12 group analysis and GMV extraction

In SPM12, two sample t-test adjusted for age and total intracranial volume (TIV) was used to assess differences between the two patients groups. A cluster forming threshold of *p*<0.001 was used and significant clusters within the explicit mask as well significant clusters in the whole brain analyses were extracted using Marsbar (http://marsbar.sourceforge.net/). Secondary analysis extracted GMV for healthy controls from significant clusters (from the analysis between patients with and without prior clinical care) to determine if patients differed from controls in these regions. Values were converted to mm^3^ using the following equation: cluster size x voxel size x beta value. The beta value extracted represents the proportion of the voxel attributed to gray matter.

### Analysis of clinical variables

Linear regression models assessed the relationship between GMV differences in those with and without prior clinical care and clinical characteristics of migraine as independent predictors. Of interest were headache clinical variables such as severity (rated as mild, moderate or severe), headache pain intensity (rated on a scale of 0-10) and headache frequency which were collected via electronic headache diaries over a 28-day period. We also collected information on psychosocial disorders (anxiety, stress and depression) and examined the relationship between GMV and psychosocial factors as independent predictors. Anxiety was measured by the Generalized Anxiety Disorder-7 (GAD-7), depression-Patient Healthy Questionnaire-9 (PHQ9) and stress-Perceived Stress Scale (PSS). All scores were modeled as both raw values and the following categories;GAD-7 was categorized as 0-4 (no anxiety), 5-9 (mild), 10-14 (moderate), 15≤ (severe). Similarly PHQ-9 was modeled categorically in bins of 5 (0-4, 5-9, 10-14, 15-19 and ≥20) to represent no depression, mild, moderate, moderately severe and severe. PSS was modeled where 0-13 is average, 14-26 is moderate stress and high stress is 27-40. Other covariates assessed as potential confounders for these models were duration of disease, sex, BMI, race, age, education and employment status. Employment status was modeled as it was reported by each individual and as a dichotomous variable (full time vs. not-full time). Final models were adjusted for age as it was correlated with disease duration, was found to be a better predictor and improved the model fit. The Chi-Square, Fishers Exact, t and Wilcoxon tests were used to assess differences in clinical and confounding variables. Secondary analyses examined demographic and GMV differences between patients and healthy controls utilizing Fishers Exact, t-tests, Wilcoxon and ANOVA tests. All statistical analyses were conducted using SAS (v.9.4, SAS Institute Inc. Cary, NC). Testing was two-sided and done at the 0.05 level of significance.

### Sample size and power

Using a two sample t-test with a two sided 0.05 level of significance, sample size of 120 patients and a group ratio of 3:1 (patients with and without prior clinical care at baseline), provided above 80 % power to detect a mean difference ranging 8-15mm^3^ between migraine patients with and without prior clinical care at baseline.

## Results

### Description of demographic and clinical profiles

There were 120 migraine patients with MRI and diagnosis data at baseline. Two patients were excluded due to abnormal brain morphology and one was excluded due to poor data quality. The final sample included 94 (80 %) migraine patients classified as having prior care for migraine and 23 (20 %) who had not. Migraine patients with prior clinical care were significantly older than those without (mean age 38 vs. 31) and reported longer median duration of disease (18 vs. 10 years) (Table 1). However, across other clinical and sociodemographic metrics both groups were comparable, being predominantly female, white, college educated, employed full time, with BMI in the normal range. Patient groups also showed no difference in headache frequency, intensity of headache pain or severity of headaches. In terms of medication use, 24 % of patients used preventatives, 55 % of patients with prior care used abortives and 67 % used over the counter (OTC) pain treatments. Patients in the prior care group often used more than one kind of medication (see details in footnote of Table 1). 60 % of patients without prior care reported using OTC pain treatments.

**Table 1:** Sociodemographic and clinical factors in EM patients with and without prior clinical care for migraine and healthy controls

Variable	Prior Medical Care /94 (80 %)	No Prior Medical Care/23 (20 %)	p value^1,*^	Healthy Controls/36	p value^2,*^
**Sex**					
Male	9 (10)	5 (22)	0.15	4 (11)	0.23
Female	85 (90)	18 (78)		32 (89)	
**Age/mean±SD**	38±12	31± 8	**0.001**	38±13	**0.02**
**Race/n (%)**^**3**^					
Black	17 (18)	6 (26)	0.14	9 (25)	0.35
White	67 (72)	12 (52)		25 (69)	
Other	9 (10)	5 (22)		2 (6)	
**Education/ n (%)**					
High School/Technical School	6 (6)	1 (4)	0.86	2 (6)	0.97
College	56 (60)	15 (65)		21 (58)	
Graduate	32 (34)	7 (30)		13 (36)	
**Employment/n (%)**					
Full-time	62 (66)	10 (43)	0.16	N/A	
Part-time	13 (14)	5 (22)			
Student	12 (13)	5 (22)			
Homemaker	2 (2)	1 (4)			
Retired	3 (3)	0			
Unemployed	2 (2)	2 (9)			
**BMI/mean±SD**	27±6	27±7	0.91	26±5	0.45
**Headache Days**					
Headache frequency/28days median (range) ^4^	8 (3-17)	9 (3-16)	0.96	N/A	
Raw Headache ^5^ Days/ median (range)	8 (3-17)	8 (3-16)	0.92	N/A	
Headache intensity/0-10	5±1.54	4±1.47	0.20	N/A	
**Headache severity**					
Mild	25 (27)	11 (48)	0.14	N/A	
Moderate	64 (68)	11 (48)			
Severe	5 (5)	1 (4)			
**Duration of disease/years Median (range**)	18 (1.5-50)	10 (1-24)	**<0.001**		
**Use of migraine/pain medications** ^6^					
Preventatives ^7^	23 (24)	N/A			
Abortives	52 (55)	N/A			
Over the Counter	63 (67)	14 (61)	N/A		
None	9 (10)	9 (39)			
**Use of non-migraine medication** ^3 8^	10 (12)	3 (14)			
PSS/ Median (range)	11 (0-28)	12 (2-32)	0.69		
GAD-7/Median (range)	1 (0-16)	1 (0-16)	0.64		
PHQ-9/Median (range)	2 (0-20)	3 (0-10)	0.42		
**Additional Chronic Pain** ^3 9^					
Yes	27 (30)	6 (30)	1.0		
No	63 (70)	14 (70)			

^1^p value for the comparison of prior care and no prior care

^2^p value for the comparison across three groups: prior care, no prior care, healthy controls

^3^Variables with missing observations

^**4**^Headache days per 28 days are adjusted headache days

^5^Raw headache days do not account for incomplete diaries

^6^Numbers for the prior care group add up to more than 94 because patients took more than one type of medication. Of the 52 patients prescribed abortives: 12 patients took abortives only, 23 took abortives and over the counter (OTC) pain killers, 8 took abortives and preventatives and 9 patients took abortives, preventatives and OTC. Of the remaining patients, 27 took OTC only, 2 patients used preventatives only, 4 patients used preventatives and OTC and 9 patients stated they had been diagnosed with migraine but did not list any current medications

^7^Patients reported using the following preventative medications: Amitriptyline, Atenolol, Candesartan, Inderal, Lamictal XR, Neurontin, Nortriptyline, Norvasc, Propranolol ER, Topamax, Venlafaxine, Verapamil

^8^There were five patients taking medication for anxiety and 2 patients taking medication for IBS, depression, and hypertension. The followings diseases all had only one patient taking medication: ADD, bipolar, heart disease, Crohn’s disease, hypothyroid, ulcers and pituitary adenoma. Data was missing on 11 patients

^9^The following pain chronic pain conditions were included: fibromyalgia, osteoarthritis, rheumatoid arthritis, chronic pelvic pain, low back pain, neck pain, temporomandibular joint disorder

*T test, Chi Square, Wilcoxon, Kruskal Wallis p value

*EM* Episodic Migraine, *PSS* Perceived Stress Scale, *GAD* Generalized Anxiety Disorder-7, *PHQ-9* Patient Healthy Questionnaire-9

There were 39 healthy controls enrolled in the study but three did not have baseline MRI data, resulting in a final sample of 36 controls. Similar to patients, controls were primarily female, white and college educated. Controls were of comparable age of patients with prior care for migraine (38 years) but older than those without prior care (31 years) (Table 1).

### MRI- GMV differences between episodic migraine patients with and without prior clinical care

**Fig. 1:**
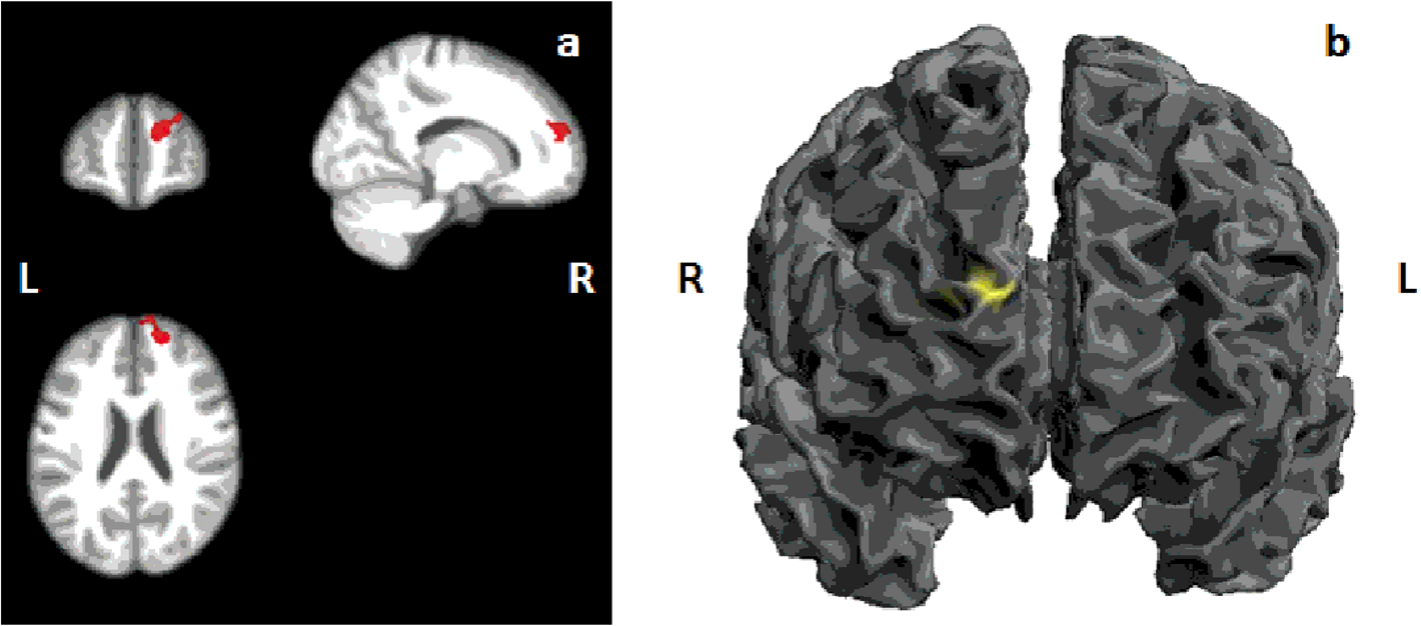
Cluster results showing region of reduced GMV in the right dorsal medial prefrontal cortex. The results are from a t-test of EM patients without prior clinical care compared to those with prior clinical care. **A** displays the cluster on the average brain created from all 117 patients. Cluster forming threshold of *p*=0.001, k=418, pFWE<0.05 at cluster-level. **B** displays the cluster on the SPM12 cortical surface. L: left; R: right

In the explicit mask analyses no significant clusters differed between the two groups. However whole brain analyses showed that patients without prior clinical care had lower GMV in one cluster located in the right dorsal medial prefrontal cortex (R DMPFC) (*p*=0.034, FWE cluster-level corrected) compared to those with prior clinical care. The average volume in patients with prior clinical care was 471.68mm^3^ compared to 435.92mm^3^ in those without. Healthy controls had an average GMV of 599±32 mm^3^ in the R DMPFC cluster. This was significantly larger than both patient groups (<0.0001). The cluster comprised 418 voxels and extends to include Brodmann Area 10 (BA10), portions of the right middle frontal gyrus (MFG), medial superior frontal gyrus and medial frontal gyrus (Fig. 1A and B). Table 2 details the size and location of the cluster as well as peak voxels within the cluster.

**Table 2:** Decreased GMV from whole brain analyses comparing EM patients with and without prior clinical care

Brain Region	Peak Voxel MNI Coordinates (x, y, z)	Cluster Size	p value
Right Dorsal Medial Prefrontal Cortex	18, 51, 20	418	**0.03**
	30, 57, 30		
	16, 45, 27		

*p value from SPM12 analyses. Analyses adjusted for age and total intracranial volume

*MNI* Montreal neurological institute, *GMV* Grey Matter Volume, *EM* Episodic Migraine

### Difference in GMV in EM patients with and without prior clinical care and the relationship with clinical characteristics

We assessed disease duration, headache frequency, severity (mild, moderate, severe), headache pain intensity, and specific psychosocial disorders associated with migraine (anxiety, stress and depression). There were no significant associations with headache clinical characteristics. However, in the exploration of psychosocial measures we observed a significant association between GMV and depression and stress. Table 3 shows the results from the linear regression models of GMV in the right DMPFC with patient group (prior care vs. no prior care) and depression/stress modeled as predictors. Both the depression and stress models show that those patients without prior clinical care have less GMV (approximately 53mm^3^) compared to those with clinical care. Consistent in both models GMV increased with increasing scores on the depression and stress questionnaire. With every point increase on the depression PHQ-9 questionnaire there was a 4.60mm^3^ increase in GMV. For the stress model there was a 2.15mm^3^ increase with every point increase on the stress PSS questionnaire. There was no interaction between group and depression or stress. (Additional File 2).

**Table 3:** Association between GMV in EM Patients and psychosocial factors

Variable	Beta Value (mm^3^)	Standard Error	p value
**Depression**			
**Prior Care (REF)**	517	20	
**No Prior Care**	-53	13	<0.001
**Depression**	4.60	1.72	0.009
**Stress**			
**Prior Care (REF)**	505.57	21.62	
**No Prior Care**	-52.68	13.45	<0.001
**Stress**	2.15	0.78	0.007

* Beta values represent the grey matter volume in the right dorsal medial prefrontal cortex in mm^3^. All models are adjusted for age; it was correlated with disease duration and was a better predictor in the model; *REF* reference level for comparison, *DMPFC* dorsal medial prefrontal cortex, *EM* episodic migraine, *GMV* Gray Matter Volume

## Discussion

Recently our knowledge of migraine pathophysiology has improved tremendously, but gaps remain of how treatment differences are reflected in brain changes. We show that though migraine patients regardless of prior clinical care, present with similar clinical and sociodemographic profiles there exists an underlying difference in brain pathology. To our knowledge this is the first study to highlight GM differences between migraine patients based on whether they have had prior care. Migraine patients who had not received prior care had less GMV in the right DMPFC compared to those with prior care. Both groups had less GMV compared to healthy controls. The cluster included portions of the right MFG, medial frontal gyrus, and medial SFG, all regions which play critical roles in migraine pathophysiology and clinical characteristics. [8, 10] The prefrontal cortex has also been shown to differ between migraine patients and healthy controls in several studies. Specifically, the left MFG has shown reduced GMV volume in migraine patients. [9, 10, 18] Further work to differentiate between subtypes of migraine patients, have used several approaches and localized the bilateral MFG and other regions in the pre-frontal cortex to classify migraine patients from other headache types as well as EM from CM. [11].

Conversely, a recent study in an Italian cohort found that regions in the frontal and temporal lobes including the R SFG, left middle temporal gyrus and right inferior frontal gyrus of migraine patients had increased GMV compared to controls and this was positively correlated with headache symptoms. [19] However in those studies, which reported reductions in GMV there were negative correlations with headache severity, duration and headache frequency. [8–10, 18] This negative association with headache clinical variables was not present in this current study, as we found no significant relationship between GMV and headache symptomology. However, there was a positive association with stress and depression. Our results can be examined in the context of these psychosocial factors and clinical findings in other pain patients. It should be noted that this relationship is likely driven by small numbers of patients with high psychosocial scores, thus the association we observe is hard to interpret. Plausible explanations are that patients with these scores may be more likely to seek clinical care, or that the interaction of headaches and psychosocial comorbidities affects the brain differently than each disorder separately.

The medial prefrontal cortex (mPFC) has been implicated in many pain disorders including IBS, fibromyalgia, back pain and migraine. [20–23] Chen et al. reported smaller frontal regions in chronic compared to EM and a negative correlation between headache frequency and frontal pole volume. [24] In cluster headache patients, GMV in the frontal pole was lower compared to those with migraine. [25] Though these works indicate that the frontopolar cortex (FPC) is important in migraine pathology, there have not been any studies in migraine patients examining how clinical care may impact brain structure. Research in other chronic pain conditions has shown that with successful treatment there is recovery of abnormal brain structure in the prefrontal cortex. In chronic back pain patients, thinning in DLPFC in patients compared to controls was normalized after treatment and was correlated with reductions in pain and physical disability. [21] Though we found that healthy controls had significantly larger GMV compared to both patient groups, the fact that those with prior care had higher GMV than those without would suggest that treatment for migraine allowed them to recover some GMV. One RCT in migraine found that baseline mPFC volume could predict the hypoalgesia response with sham acupuncture after 8 weeks (mediated by decreased anxiety). [20] Fettes et al. showed abnormal functional connectivity in the medial and lateral FPC in treatment-nonresponsive major depressive disorder patients which was correlated with symptom severity. [26].

This gives some insight into the role that the mPFC plays not only in migraine but in the way it interacts with psychological disorders in migraine and subsequent migraine improvement. The interaction between migraine and co-morbid psychological disorders is of growing interest due to the prevalence of these disorders in the migraine population. A recent imaging study looked at differences in intrinsic activity measured by amplitude of low-frequency fluctuation (ALFF) in migraineurs with and without co-morbid depression. Researchers found significant main effects of migraine and depression in the left mPFC where there was increased ALFF associated with both disorders. It was concluded that these findings may indicate a common therapeutic target for migraineurs with co-morbid depression. [27].

In light of previous studies our results add to a growing body of work which highlight the mPFC as not only an important role in chronic pain, but an important region in the treatment of pain patients. We show that this region may also adversely affected in patients who do not receive care for their migraine headaches. However, we do have some limitations. In our sample almost 20 % of patients had never received prior clinical care for their migraine. Compared to population based studies where less than half of migraine patients often report receiving medical consultation for their condition [3, 4] our sample of patients had a much higher proportion of diagnosed patients (80 %). This is likely due to the fact that a major component of the recruitment strategy was targeted to headache clinics. This means that our sample was not representative of the wider migraine community where diagnosis of migraine is less common. It is possible that this difference in sample size could bias our results, either falsely finding a difference where there is none, or occluding additional differences which may be important to address in those patients who have not received care. However we did conduct power analyses which showed that even with a 3:1 ratio of patients between groups, we were powered above 80 % to detect differences in GMV between patients with and without prior care. Our patients also had comparable sociodemographic and clinical profiles, regardless of prior care status which does address some concerns regarding differences in sample size. While most epidemiological literature has found difference in socioeconomic and headache severity status between those who seek care and those who do not, this was not present in our study. Given that we were unable to directly assess socioeconomic status, and there was no association between headache factors and GMV, there may be some unmeasured confounding in the sample. The demographic similarity between groups was likely because our patients were recruited for a clinical trial and will not be representative of the general population of migraine patients. Patients were classified into groups based on patient report of migraine diagnosis and migraine medication. One drawback to this approach was the difficulty in collecting information on the duration of therapy in patients. Given that the data was based on patient recall and not review of a medical record, patients provided information on start dates in a manner that was not useable for analysis or to estimate an average duration for the sample. Since we found differences between patients based on prior care (including medication use) it is possible that duration of treatment is an important factor in our observed findings. Because we are unable to adjust for this in analysis we cannot elucidate differences between those who recently started therapy and those who had been on treatment for a long time. Further, we cannot determine how they differ from those without prior care. Finally, though preventative and abortive treatments work very differently and play different roles, there was substantial overlap between medication use in patients with prior care and thus differences between these two types of treatments could not be elucidated. Additionally there could be some occlusion of our results given patients in both groups used over the counter (OTC) pain medication for the treatment of acute migraine. However use of OTC between groups was similar (67 % vs. 61 %) and thus the use of OTC in the no prior care group likely did not have an effect on the results.

We do however present a study with several strengths. Though the proportion of undiagnosed patients was smaller than expected, we observed a much larger difference in GMV between patients than anticipated. Power analyses were based on expected differences of 8-15mm^3^ and we report a difference of almost 36mm^3^ between patient groups. This is also the first study which exclusively compares GMV differences between migraine patients, based on prior clinical care and though it has some limitations, it provides a foundation upon which further research can be built. Taken together, research in several patient populations, points to the common theme that abnormal structure and function in several regions in the prefrontal cortex (DLPFC, mPFC) may both predict treatment success and be altered with successful treatment.

## Conclusions

Our results provide further evidence that EM patients who are not receiving care may have an added burden associated with this lack of care even though clinically they may present the same. Given these findings it is clear that efforts to improve access to migraine treatment need to be expanded. Furthermore, these results indicate that some patients may need different treatment options to address this baseline deficit. Future studies should aim at following treatment naïve patients to determine if they take longer to regain GMV over time and if they require either different or more intense therapy to reach treatment success.

## Supplementary information

Additional file 1: **Supplemental Fig. 1. **Explicit mask used for region of interest analyses overlaid on average brain of all participants. Brain image showing the regions of the brain included in explicit mask analyses. Mask includes the bilateral SFG, DLPFC, insula, and cingulate cortex.

Additional file 2 **Supplemental Fig. 2. **Scatter plot of right dorsal medial prefrontal gyrus GMV with stress (PSS) score (a) and depression (PHQ-9) score (b) in EM patients with (blue) and without (red) prior clinical care. Plot of the relationship between GMV in the right DMPFC and psychosocial factors to show how GMV varies with scores.

## Data Availability

The datasets used and/or analysed during the current study are available from the corresponding author on reasonable request.

## Abbreviations

ACC: Anterior Cingulate Cortex
AMPP: American Migraine Prevalence and Prevention Study
BMI: Body Mass Index
CAT12: Computational Anatomy Toolbox
CM: Chronic Migraine
CSF: Cerebrospinal Fluid
DLPFC: Dorsolateral prefrontal cortex
DMPFC: Dorsal Medial Prefrontal Cortex
EM: Episodic Migraine
FOV: Field of View
FPC: Frontopolar Cortex
FWE: Family wise error
GM: Gray Matter
GMV: Gray Matter Volume
GAD-7: Generalized Anxiety Disorder-7
HHF: Health History Form
MFG: Middle Frontal Gyrus
MNI: Montreal Neurological Institute
MRI: magnetic resonance imaging
OTC: Over The Counter
PHQ-9: Patient Healthy Questionnaire-9
PSS: Perceived Stress Scale
SFG: Superior Frontal Gyrus
SPM12: Statistical Parametric Mapping
TE: Echo Time
TIV: Total Intracranial Volume
TR: Repetition Time
VBM: Voxel Based Morphometry
WM: White Matter
