# Supplementary material for: Differences in gray matter volume in episodic migraine patients with and without prior diagnosis or clinical care: a cross-sectional study
Source: J Headache Pain. 2021 Oct 23;22(1):127. doi: 10.1186/s10194-021-01340-5 (PMC8542322; doi:10.1186/s10194-021-01340-5)
Supplement: Supplementary file 1 — Supplemental Fig. 1. Explicit mask used for region of interest analyses overlaid on average brain of all participants. Brain image showing the regions of the brain included in explicit mask analyses. Mask includes the bilateral SFG, DLPFC, insula, and cingulate cortex. [file 10194_2021_1340_MOESM1_ESM.docx]

**
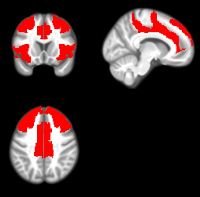
**

**Supplemental Figure 1: Explicit mask used for region of interest analyses overlaid on average brain of all participants.** *Mask includes the bilateral SFG, DLPFC, insula, and cingulate cortex.
