# Supplementary material for: Differences in gray matter volume in episodic migraine patients with and without prior diagnosis or clinical care: a cross-sectional study
Source: J Headache Pain. 2021 Oct 23;22(1):127. doi: 10.1186/s10194-021-01340-5 (PMC8542322; doi:10.1186/s10194-021-01340-5)
Supplement: Supplementary file 2 — Supplemental Fig. 2. Scatter plot of right dorsal medial prefrontal gyrus GMV with stress (PSS) score (a) and depression (PHQ-9) score (b) in EM patients with (blue) and without (red) prior clinical care. Plot of the relationship between GMV in the right DMPFC and psychosocial factors to show how GMV varies with scores. [file 10194_2021_1340_MOESM2_ESM.docx]

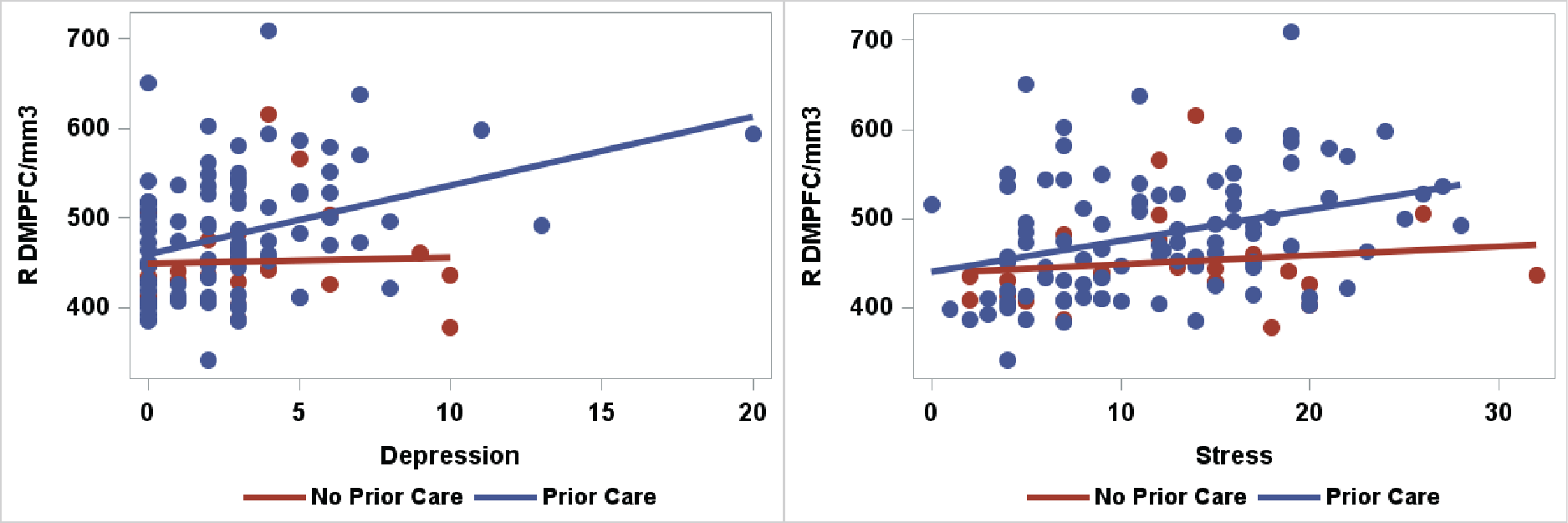


**b**

**a**

**Supplemental Figure 2: Scatter plot of right dorsal medial prefrontal gyrus GMV with stress (PSS) score (a) and depression (PHQ-9) score (b) in EM patients with (blue) and without (red) prior clinical care.**
